# Cleft lip and Palate: A 30-year Epidemiologic Study in North-East of Iran

**Published:** 2015-01

**Authors:** Hamidreza Kianifar, Nadia Hasanzadeh, Arezoo Jahanbin, Atefeh Ezzati, Homa Kianifar

**Affiliations:** 1 *Department of Pediatrics,Ghaem Hospital, School of Medicine, Mashhad University of Medical Sciences, Mashhad, Iran.*; 2 *Dental Material Research Center, School of Dentistry, Mashhad University of Medical Sciences, Mashhad, Iran.*; 3 *Department of Orthodontics, School of Dentistry, Mashhad University of Medical Sciences, Mashhad, Iran.*; 4 *Dental Research Center, School of Dentistry, Mashhad University of Medical Sciences, Mashhad, Iran.*; 5 *Faculty of Medicine, Mashhad University of Medical sciences, Mashhad, Iran.*

**Keywords:** Cleft lip, Cleft palate, Epidemiology, Incidence, Iran

## Abstract

**Introduction:**

Cleft lip and palate are among the most common congenital anomalies worldwide. This study was conducted in order to explore the incidence and related factors of cleft lip and/or palate (CL/P) among live births in Mashhad, North-Eastern Iran.

**Materials and Methods:**

In this cross-sectional study, records of 28,519 infants born between March 1982 and March 2011 at three major hospitals in Mashhad were screened for oral clefts. Clinical and demographic factors relating to diagnosed cases, including birth date, gender, birth weight, maternal age, number of pregnancies, type and side of cleft and presence of other congenital anomalies were recorded for analysis.

**Results:**

The overall incidence of CL/P was 1.9 per 1,000 live births. Cleft lip associated with cleft palate (CLP) was the most prevalent type of cleft (50%), followed by isolated cleft lip (35.2%) and isolated cleft palate (14.8%). A total of 92.6% of oral clefts were bilateral and 5.5% were located on the right side. In addition, clefts were found to be more common in male than female births (male/female ratio=2.3). The rate of associated congenital anomalies in CL/P newborns was 37%. No significant differences were observed in the incidence of oral clefts across three decades of study; except for CLP which was significantly more prevalent between 2002–2011 (P=0.027). There were no significant differences with regard to season of birth, associated anomalies or maternal age of affected newborns in the three time periods of the study. Furthermore, maternal age and number of pregnancies were not significantly different among the three types of cleft (P=0.43 and P=0.91, respectively). Although the mean birth weight of patients affected with isolated cleft palate was considerably lower than that of the other two types of cleft, the difference was not statistically significant (P=0.05).

**Conclusion:**

This study indicates a frequency of CL/P close to the findings in East Asian countries and higher than some previous reports from Iran, European and American countries. Ethnicity-related genetic factors may have a role in the conflicting results obtained from different populations.

## Introduction

Cleft lip and/or cleft palate (CL/P) are the most common congenital anomalies in the oral and maxillofacial region and exhibit a multi-factorial etiology, including environmental factors and genetic background (1,2). Not only the facial appearance, but also functions such as hearing, phonation, mastication, deglutition, and ventilation are altered by this malformation (3). Treatment of patients affected with CL/P is a multidisciplinary process from birth into adolescence and adulthood. Therefore, CL/P may inflict a large burden on the financial, emotional, and psychosocial status of affected individuals and their family members (4,5).

In general terms, the incidence of CL/P is estimated to be between 0.8 and 1.7 cases per 1,000 live births (6,7). The incidence of clefts may be influenced by geographic, racial and socioeconomic factors (8). Several studies have demonstrated that the incidence is highest among Asians, followed by Caucasians, and lowest in people of African descent (9-12). Another investigation demonstrated that internationally, during the period 2000 to 2005, the overall prevalence of cleft lip with or without cleft palate was 9.92 per 10,000 live births. In this study, the prevalence of cleft lip was 3.28 per 10,000, and that of cleft lip and palate was 6.64 per 10,000 (13). Most of the epidemiological studies on CL/P have been conducted in the USA, Europe or Asian countries. In Iran, the overall incidence of oral clefts was reported to be 1.03 per 1,000 births (14). Although there have been a few published epidemiological investigations concerning oral clefts in Iran (15-17) there is a lack of information about the prevalence of cleft lip and palate in North-Eastern Iran. Considering the importance of obtaining accurate estimates of the frequency and other epidemiological features of oral clefts, this study was conducted in order to assess the incidence and related factors of CL/P among live births in Mashhad, North-Eastern Iran.

## Materials and Methods

In this retrospective cross-sectional study 28,519 records were randomly selected (using a simple random method) from documented files of infants born between March 1982 and March 2011 at three major hospitals (Ghaem, Imam Reza and Ommolbanin) in Mashhad, North-Eastern Iran. These hospitals are the biggest hospitals in Mashhad and their delivery rate is much higher than other hospitals, among patients from all over the city and from different strata of society. 

This study was approved by the Ethics Committee of Mashhad University of Medical Sciences. The inclusion criteria included live births at 40 weeks of gestation. Only mothers with Iranian ethnicity were included in the study. The demographic data included birth date, gender, birth weight, age of mother, number of pregnancies, history of abortion, type and side of oral cleft and presence of other congenital anomalies. 

The collected data were processed using the statistical package for social science, version 11.0 (SPSS Inc, Chicago, IL, USA). Descriptive statistics, Chi-square test and analysis of variance were used for data analysis. A P-value less than 0.05 was considered statistically significant.

## Results

From a total of 28,519 cases, 54 children with CL/P were born between March 1982 and March 2011. The overall incidence of CL/P was 1.9 per 1,000 live births. Distribution of newborns affected with CL/P, according to the type and side of cleft is presented in Table 1. Cleft lip associated with cleft palate (CLP) was the most prevalent type of cleft (50%), followed by isolated cleft lip (35.2 %) and isolated cleft palate (14.8%). A total of 92.6% of the oral

clefts were bilateral and 5.5% were located on the right side (Table. 1).

**Table 1:** Distribution of clefts by type and side

**Type of Cleft**	Unilateral				**Bilateral**		**Total**	
	Right		Left					
	n	%	n	%	n	%	n	%
**CL**	2	10.5	1	5.3	16	84.2	19	35.2
**CP**	0	0	0	0	8	100.0	8	14.8
**CLP**	1	3.7	0	0	26	96.3	27	50.0
**Total**	3	5.5	1	1.9	50	92.6	54	100

CL: cleft lip; CP: cleft palate; CLP: cleft lip and palate.

According to Table 2, no significant differences were observed between the newborns across the three decades regarding the incidence of cleft lip or isolated cleft palate (P=0.319 and P=0.386, respectively). In contrast, the incidence of cleft lip with cleft palate in cases born between 2002 and 2011 was significantly higher than that in the first two decades (1982–1991, 1992–2001) (P=0.027).

**Table 2:** Comparison of cleft types among three time periods

**Type of cleft**	Time period							
	1982-1991		1992-2001		2002-2011			
	n	%	n	%	n	%	χ^2^	P
**CL**	6	31.6	3	15.8	10	52.6	2.287	0.319
**CP**	2	25.0	3	37.5	3	37.5	1.905	0.386
**CLP**	4	14.8	1	3.7	22	81.5	7.219	0.027*
**Total**	12	22.2	7	13.0	35	64.8		

CL: cleft lip; CP: cleft palate; CLP: cleft lip and palate.

* P<0.05.

Table 3 shows the sex distribution, season of birth, and associated anomalies in the affected newborns. Oral clefts were found to be more common in male than female births (male/female ratio=2.3). However, no significant differences were observed between genders across the three decades

 (P=0.513, Table. 3). The rate of associated congenital anomalies in CL/P newborns was 37%. A Chi-square test did not reveal significant differences regarding the season of birth or concomitant anomalies between affected newborns across the three decades

 (P=0.372, P=0.917, respectively) (Table.3).

**Table 3:** Sex distribution, season of birth, and associated anomalies in the affected neonates

**Related factors**	Time period								
		1982-1991		1992-2001		2002-2011			
		n	%	n	%	n	%	χ^2^	P
**Sex**	Male	10	83.3	5	71.4	23	65.7	1.335	0.513
	Female	2	16.7	2	28.6	12	34.3		
**Associated anomalies**	Yes	4	33.3	3	42.9	13	37.1	0.172	0.917
	No	8	66.7	4	57.1	22	62.9		
**Season of birth**	Spring	1	8.3	3	42.8	13	37.1	6.477	0.372
	Summer	5	41.7	2	28.6	13	37.1		
	Autumn	5	41.7	1	14.3	5	14.3		
	Winter	1	8.3	1	14.3	4	11.5		

In total, the mean age for mothers of CL/P infants was 25.72 (SD=7.14). The mean number of pregnancies was 2.79 (SD=2.54) and the mean number of abortions was 0.45 (SD=0.68). Moreover, the mean birth weight of CL/P infants was 2.11 kg (SD=1.26).

As shown in Table 4, significant differences were observed among three decades with respect to number of abortions (P=0.014), number of pregnancies (P<0.001), and newborn weight (P<0.001). However, maternal age was not significantly different among the three time intervals (P=0.060).

In general, no statistically significant association was detected between CL/P type and neonate gender, season of birth or presence of other congenital anomalies (Table. 5). 

According to Table 6, maternal age and number of pregnancies were not significantly different among the three types of cleft (P=0.43 and P=0.91, respectively). Although the mean birth weight of patients affected with isolated cleft palate was considerably lower than the other two types of cleft, the difference was not statistically significant (P=0.05, Table. 6). 

## Discussion

This cross-sectional study was carried out to explore the prevalence of oral clefts over a 30-year period in Mashhad, North-Eastern Iran. The overall incidence of CL/P was found to be 1.9 per 1,000 live births. Previously, Farhud et al (18) and Khazaei et al (14) have reported an overall incidence of 1 cleft per 1,000 births in Iran. Likewise, in an epidemiologic study by Rajabian et al (19), the prevalence of oral clefts in Iran was 1.03 per 1,000 births. Yassaei et al (16) reported a prevalence of 0.86 per 1,000 live births in Yazd located in the central part of Iran. Moreover, prevalence rates of 0.97, 1.01, and 0.80 were reported for CL/P in the North, North-West and South-West of Iran, respectively (15,17,20). In Tehran, the capital of Iran, the overall incidence of CL/P from 1998 to 2005 and from 2004 to 2008 was reported to be 2.14 and 1.79 per 1,000 live births, respectively (21,22). However, Taher et al (23) reported a prevalence of 3.73 per 1,000 live births in Tehran from 1983–1988. This high incidence of CL/P was attributed to the chemical gas agents used during the Iran-Iraq conflict 23). On the other hand, Jahanbin et al (24) reported no significant difference in the incidence of oral clefts between the period of the Iran-Iraq war (1982–1987) and recent years.

Internationally, incidence rates of 1.94 per 1,000 in the Philippines (25), 1.81 per 1,000 in Korea (26), 1.91 per 1,000 in Pakistan (27), 1.39 per 1,000 in Jordan (28), 1.53 per 1,000 in Scotland (29), 0.34 per 1,000 in Africa (30) and 0.77 per 1,000 in the USA (12) have been reported.

It seems that the incidence of CL/P in North-Eastern Iran is similar to that in Pakistan and some Asian countries, but higher than among individuals of Caucasians and African descent. Variations in genetic susceptibility and environmental factors may be the cause of this discrepancy in the rate of CL/P among different populations (31).Furthermore, there is a large variation in the distribution of different types of oral clefts. The present survey in North-Eastern Iran shows the frequency of isolated CL (35.2%), isolated CP (14.8%) and CLP (50%). These results coincide with those found among some other populations of Iran (17,19). However, a study from Tehran (the capital of Iran) has reported these figures at 12%, 36%, and 52%, respectively (22). While the most common type of cleft reported in Scotland was isolated cleft palate (29), isolated cleft lip was more common than other facial clefts in Nigeria (30). In the present study, the frequency of oral clefts was not significantly different across the three decades, except for CLP which was more prevalent in cases born between 2002–2011 than in the first two decades of the study. In terms of the side of the cleft, contrary to other reports (19,31), we found right predominance among unilateral cases.

The present study showed a nonsignificant gender difference in the frequency of oral clefts, with a male-to-female ratio of 2:3. The male dominance in the prevalence of CL/P is also shown in other reports from Iran (17,31), Korea (26) and Europe (29). 

In addition, this investigation showed no significant differences with regard to season of birth, associated anomalies and maternal age of affected newborns in the three time periods of the study. Likewise, Yassaei et al (16) reported no significant differences in the occurrence of CL/P regarding season of birth or maternal age. Jahanbin et al (32) also found there was no significant seasonal trend in birth dates of children affected with clefts in Mashhad.

The overall percentage of affected newborns with associated anomalies was higher than that in a previous study in Iran (15), but was similar to studies conducted in Europe (29,33).

In the present study, the mean birth weight of CL/P cases born between1992–2001 in Mashhad was significantly lower than the weight of affected newborns in the other two decades. However, the frequency of cleft lip and palate during these years was lower compared with other time periods. Besides, the prevalence of other types of cleft was not significantly different among the three decades. These findings do not support an association between low birth weight and oral clefts. Conversely, Lei et al (2) showed a significant relationship between low birth weight of <1.5 kg and facial cleft deformities in Taiwan. The possibility of the influence of the oral cleft on birth weight needs to be clarified in further studies.

Our study has some limitations. The database provided no information on the consanguinity of parents, maternal health or peri-conceptual conditions, which may play a role in the development of orofacial clefts (7,11). Additional demographic factors such as socioeconomic status and area of residence, which have been associated with different prevalence rates (2), were also not recorded in the database.

## Conclusion:

The overall prevalence for congenital cleft deformities in North-Eastern Iran was 1.9 per 1,000 live births, close to the findings in East Asian countries and higher than some previous reports from Iran, European and American countries. Ethnicity-related genetic factors may have a role in the frequently conflicting results regarding prevalence of cleft deformities in different populations. Therefore integrating genetic analysis into epidemiologic studies will be necessary in future studies.
